# Recent advances on polymeric hydrogels as wound dressings

**DOI:** 10.1063/5.0038364

**Published:** 2021-02-16

**Authors:** Zheng Pan, Huijun Ye, Decheng Wu

**Affiliations:** Department of Biomedical Engineering, Southern University of Science and Technology, No. 1088 Xueyuan Avenue, Nanshan District, 518055 Shenzhen, Guangdong Province, China

## Abstract

Severe hemorrhage is a leading cause of high mortality in critical situations like disaster, accidents, and warfare. The resulting wounds could induce severe physical and psychological trauma to patients and also bring an immense socio-economic burden. Hence, rapid hemostasis and wound healing techniques have become critical initiatives for life-saving treatment. Although traditional methods relying on bandages and gauzes are effective in controlling hemorrhage, they suffer from several limitations: nonbiodegradability, being susceptible to infection, being unsuitable for the irregular wound, secondary tissue damage, and being almost ineffective for wound healing. Owing to the merits of high porosity, good biocompatibility, tunable physicochemical properties, and being beneficial for wound healing, hydrogels with excellent performance have drawn intensive attention and numerous novel effective hydrogel dressings have been widely developed. In this Review, after introducing some commonly used strategies for the synthesis of hydrogels, the most recent progress on polymer-based hydrogels as wound dressings is discussed. Particularly, their hemostasis, antibacterial, and biodegradation properties are introduced. Finally, challenges and future perspectives about the development of hydrogels for wound dressings are outlined.

## I. INTRODUCTION

The widespread use of high-speed and high-energy weapons in modern warfare has led to an increasing incidence of explosive injuries. For instance, during the Iraq and Afghanistan wars, the proportion of explosive injuries in the U.S. military was as high as 70%.^1,2^ Compared with conventional weapons, the explosive weapons are more prone to induce serious soft tissue trauma, like perforation and irregular wounds, which are usually accompanied by massive hemorrhage, wound infection, and even severe complications. In various accidents, traumatic bleeding is also one of the most common injuries, and uncontrollable hemorrhage is considered as the primary cause of death at the scene.^3^ Even if the injured person could be sent to the hospital for rescue, excessive loss of blood during prehospital treatment would still pose a high risk of death or serious complications later.^4,5^ Massive bleeding seriously endangers the lives, but most of the casualties caused by hemorrhage could be avoided. It is reported that the first hour since injury is the “golden time” for rescue lives, and the initial 10 min (so-called platinum ten) is even more precious, during which time the bleeding should be fully controlled so as to ensure survival of the victims.^6^

After blood clotting, the healing of the damaged tissues and organ structures is another serious challenge for doctors and patients. As a complex and dynamic process, wound healing can be divided into four partially overlapping steps: (1) hemostasis; (2) inflammation; (3) proliferation (angiogenesis, granulation, and re-epithelialization); and (4) tissue remodeling.^7–9^ Infection, necrosis, and second bleeding during the healing process would prolong the treatment time and also lead to disability or even sometimes death.^10–12^ Therefore, rapid hemostasis, prevent infection, and promote repair are pivotal for life-saving treatment in the battlefield and accident.

Currently, conventional dressings like gauzes and bandages are still widely used in the clinic. However, they have an unsatisfactory hemostatic performance for arterial ruptures and wounds with irregular, deep, narrow shapes. In addition, they need long-term treatment, are unsuitable for inherently complicated procedures, easily adhere to desiccated wound surfaces, and are required to be surgically and mechanically removed from the damaged area, which inflict serious secondary damage on patients.^13–16^ A favorable dressing should fulfill the following characteristics: it should (1) easily accommodate complex wound contours and volumes, (2) possess mechanical protection, (3) keep a moist environment, (4) present ideal permeability of gases, (5) be capable of absorbing exudates, (6) protect the wound from the infection of bacteria, (7) be easily and atraumatically changed and removed, (8) be able to be stored for a long time in extreme environments, (9) be costly/commercially acceptable, (10) be light in weight, and (11) be nontoxic, nonallergic, biocompatible, biodegradable, and elastic.^17–21^

**FIG. 1.**
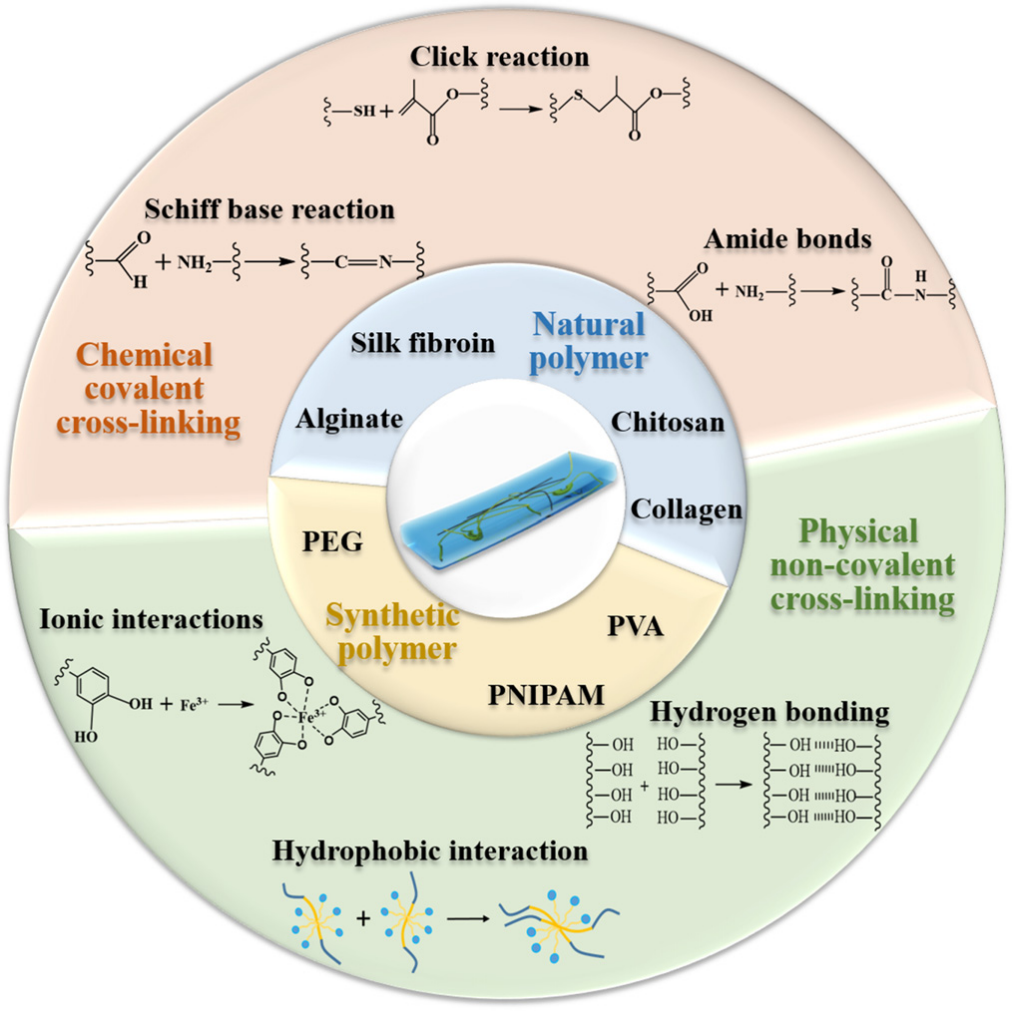
Crosslinking mechanisms and raw materials for various hydrogel wound dressings.

With the growing demand, developments and manufactures of novel wound dressings with high performance have become a research focus in the field of medical materials, among which hydrogel is found to satisfy most of the aforementioned criteria for nursing wounds.^22–24^ Hydrogel is a 3D network composed of hydrophilic polymers, which can absorb and swell in water.^25^ Due to the highly mimic natural extracellular matrix (ECM) properties, hydrogel has been extensively utilized in pharmaceutical and biomedical applications. In terms of wound dressings, hydrogel could not only form a physical barrier and remove excess exudate but also encapsulate bioactive molecules and provide a moisture environment that promotes the wound healing process.^26,27^ In addition, the injectable hydrogel would also be able to completely fulfill the irregular shaped wound and deal with deep bleeding wound more efficiently.^28,29^ Due to the numerous merits of hydrogels, a series of commercial hydrogels as wound dressings have been emerged, such as Algisite M, Tegaderm™ hydrocolloid dressing, Evicel^®^, and Coseal^®^. The composition and applications of these products are summarized in Table I. Despite their good wound healing performance, there exist some deficiencies, including high cost, harsh storage conditions, inability to provide adequate mechanical protection, and poor permeability of gases. Since the demand for higher performance dressings, novel hydrogel dressings with multifunctional properties (e.g., antibacterial ability, biodegradability, responsiveness, and injectability) received increasing attention in the field of wound dressings in recent years. Herein, the Review aims to provide a state-of-art overview about the development of hydrogel for wound dressings, including the general synthetic strategies and its raw materials (shown in Fig. 1), and their advantages and disadvantages are shown in Tables II and III, respectively.

**TABLE I.** The composition and applications of commercial wound dressings.

Brand	Composition	Applications
Algisite M	Alginate	Abrasions
		Burns
		Diabetic foot ulcers
		Surgical wounds
Tegaderm™ hydrocolloid dressing	Glucose	Partial- and full-thickness dermal ulcers
		Superficial wounds and abrasions
		Superficial and partial-thickness burns
Evicel^®^	Fibrinogen	Clinical wounds
		Abrasions
		Superficial and partial-thickness burns
Coseal^®^	PEG	Massive bleeding wounds

**TABLE II.** Advantages and disadvantages of various synthetic strategies of hydrogel wound dressings.

Cross-linking methods		Advantages	Disadvantages
Chemical covalent cross-linking	Schiff-based reaction	1. Forming strong bonds	1. Almost all need toxic agents
	Click reaction		
			2. Needs washing to remove the residual
	Amidation reaction		
Physical noncovalent cross-linking	Ionic interaction	1. Safe	1. Lower degree of cross-linking
			2. Lower energy of bonds
	Hydrogen bonding		
		2. Less toxic than chemical agents	3. May alter the properties of the hydrogels
	Hydrophobic interaction		4. Inability to control the progress of the reaction

**TABLE III.** Advantages and disadvantages of various raw materials of hydrogel wound dressings.

Raw materials		Advantages	Disadvantages
Natural polymers	Collagen (COL)	1. Low inflammatory response	1. Lower mechanical properties of hydrogels
	Chitosan (CS)	2. High biodegradability	2. Performances of different batches greatly vary
	Silk fibroin (SF)	3. Fairly low cost	3. Difficult to modify
	Alginate	4. Mostly good for healing	
		5. Rich functional groups	
Synthetic polymers	PEG	1. Stably remained at injury site up	1. Lower biocompatibility
	Poly(vinyl alcohol) (PVA)	2. Easy to modify	2. Relatively high cost
	PNIPAM	3. Strong mechanical properties	3. Almost all require modification
		4. High purity	

## II. SYNTHESIS OF HYDROGELS

Hydrogels are formed by cross-linking techniques, which can be categorized into physical,^30^ chemical,^31^ enzymatic,^32^ and irradiated,^33^ cross-linking methods. The types of “bonds” and cross-linking methods have different advantages and disadvantages, which could also determine the physicochemical properties of hydrogels. In spite of many works about investigation of the cross-linking techniques being reported, several aspects in the process of forming a hydrogel are poorly understood and the structure and aggregation mode of hydrogels remain a research area with intense interest. In this part, various fabrication techniques of hydrogel dressings were summarized, including chemical covalent bonds, physical noncovalent coordination, and combination of chemical and physical interactions.

### A. Chemical covalent cross-linking

In considering biomedical applications of hydrogels, the chemical reaction involved should not damage the biopharmaceuticals or cells. In this chapter, Schiff base reaction, click reaction, and amide bond reaction are discussed for the preparation of hydrogels. All these gelation progress driven by chemical reactions because they involve the formation of new covalent bonds or chemical changes during the gelation.

#### 1. Schiff base reaction (imine bonds)

An imine bond is a carbon–nitrogen double bond in a compound, which is formed by a Schiff base reaction with nucleophilic attack of the amine group to the aldehyde or ketone group.^34^ The Schiff base reaction has widely emerged in the biomedical field due to its mild reaction conditions and higher reaction rates. Moreover, the formed imine bonds are reversible with the changes of the pH value, which could be utilized to design self-healing materials.^35^

It is well known that there are so many amino groups on the CS backbones, so it is often applied to prepare hydrogel dressings through Schiff base reaction. Huang *et al.*^36^ developed a nanocomposite hydrogel (CSH) dressing, which is cross-linked by dynamic Schiff base reaction between the amine group of carboxymethyl chitosan (CMC) and aldehyde group of dialdehyde-modified cellulose nanocrystal (DACNC). The CMC/DACNC hydrogel was endowed self-healing and injectable ability due to the existence of reversible imine covalent bonds. On the other hand, the hydrogel that would decompose in amino acid solution could be observed. In detail, the stability of Schiff base reaction could be destroyed by amino acids. As a free amino acid, glycine containing amine groups could also react with DACNC. Simultaneously, as a competitor, the amine groups in free amino acid have more reactivity than the amine groups on CMC in the reaction of aldehyde groups and amine groups. Thus, the collapse and solution of CMC/DACNC hydrogels happened in amino acid solution.

**FIG. 2.**
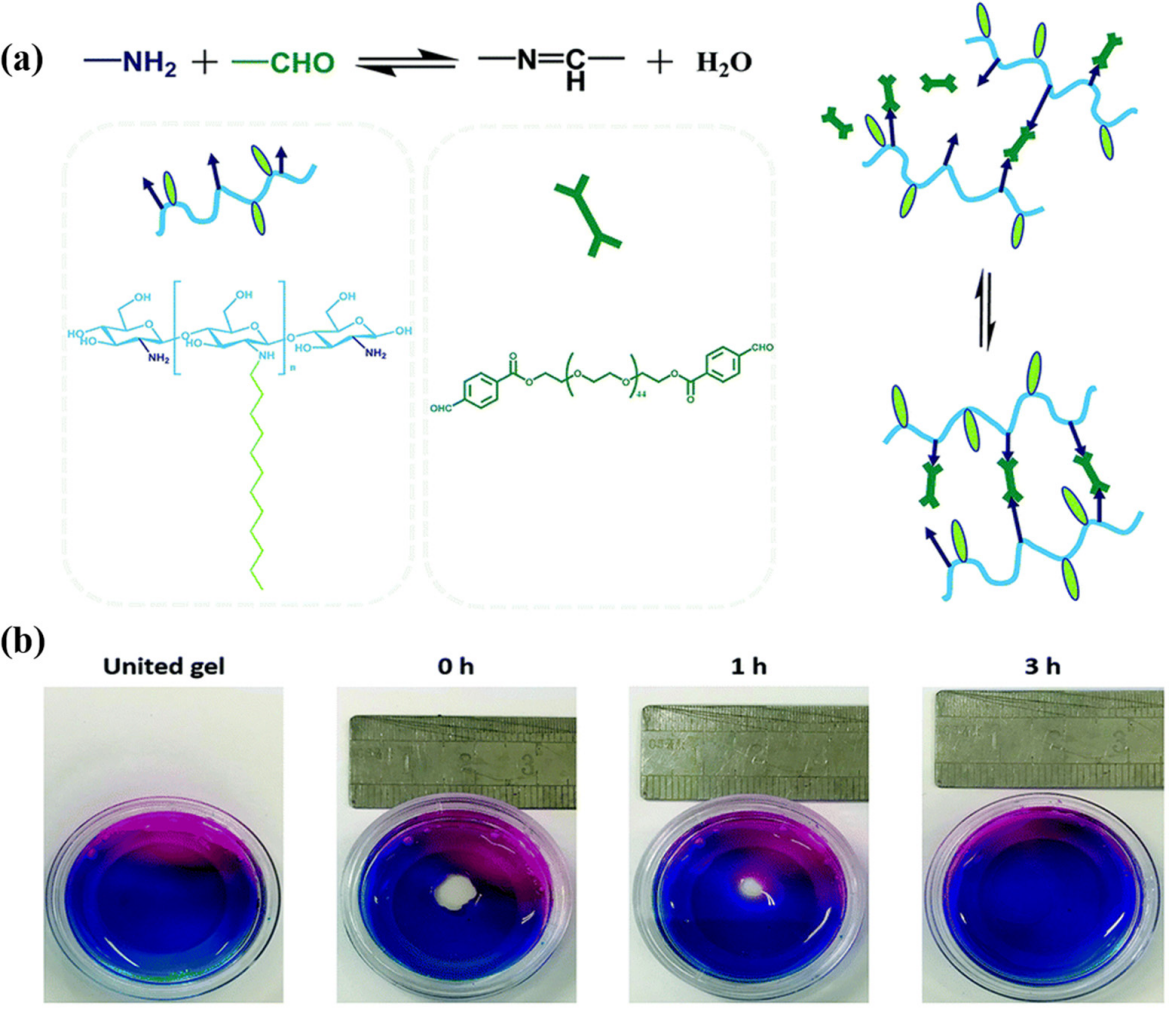
(a) Schematic of the gelation process via mixing of the dodecyl-modified chitosan (FCS) and the dialdehyde functionalized PEG (PEG-CHO) solutions. (b) Self-healing behavior of HG1 vs time. Reproduced with permission of from Yu *et al.*, J. Mater. Chem. B **8**, 3908–3917 (2020). Copyright 2020 Royal Society of Chemistry, Clearance Center, Inc.^39^

The Schiff base reaction also occurs between other polysaccharides. Liu *et al.*^37^ prepared a wound dressing via modified hyaluronan (HA) and ε-polylysine (EPL). Interestingly, because of the high positive charge density of EPL and existence of HA pieces, this dressing can not only effectively kill Gram (+) and (−) bacteria but also extremely accelerate the wound healing. Certainly, Schiff base reactions also occur between the aldehyde group and amino group introduced by grafting,^38^ PEG often appears in related articles and has been provided with stimuli-responsive properties and great mechanical properties (Fig. 2).^39,40^ It should be noted that the residual small molecular cross-linking agents containing the aldehyde group, such as glutaraldehyde, should be removed thoroughly because aldehyde groups are poisonous for cells and would trigger an inflammation response in organisms.^41^

#### 2. Click reaction

In general, the click chemistry defined by Kolb and co-workers is referred to a certain type of reaction that has the advantages of high yield, simple reaction conditions, easy availability of raw materials and reaction reagents, harmless secondary products, and fast synthetic reaction.^42^ Thiol-ene click reaction, as a kind of click chemistry, could be triggered by UV light or heat without the addition of catalysts and complete in a very short time.^43^ Due to its highly reactive efficiency, the thiol-ene click reaction has been popularly applied for preparing *in situ* hydrogels.

Shi and his co-worker^44^ had prepared a hyaluronan–bisphosphonate (HA–BP) hydrogel and applied for wound dressings. The HA–BP hydrogel was obtained from the UV-light initiated click reaction between the HA-maleimide derivative (HA-Mal) and the BP-acrylamide derivative (SH-BP). The HA–BP hydrogel is suitable for injecting, molding, or printing, because of the inherent shear-thinning and self-healing properties. For higher reaction yield and controllability, more mild and chemo-selective thiol-ene click reaction was selected. In detail, the HA-Mal was synthesized by the coupling reaction of the carboxyl groups of HA and the amino group of *N*-(2-aminoethyl) maleimide. However, the thiolated BP (SH-BP) was obtained through Michael addition reaction of BP-acrylamide and excess of dithiothreitol (DTT). Finally, the novel HA–BP derivative was fabricated via chemo-selective click reaction between HA-Mal and SH-BP. DTT frequently acts as a cross-linking agent in the process of fabricating hydrogels via thiol-ene click reaction. Qin *et al.*^45^ prepared PVA-norbornenes (PVA-NB) hydrogels through click reaction of PVA-NB and DTT. For the hemostatic application, TRAP6-presenting hydrogel particulates (PVA-TRAP6-P) were further fabricated by PVA-NB hydrogel particulates (PVA-NB-P) with thrombin-receptor-agonist-peptide-6 (TRAP6). Experimental results demonstrated that the novel hydrogel can effectively shorten the clotting time (CT) to about 50%. As another unsaturated bond, alkynyl groups have also been used to make hydrogels via thiol-yne click reaction.^46^

Except the thiol click reaction, the Diels–Alder (DA) click reaction is a promising type as long as addressing the issue that relies on high temperature (>100 °C) stimulation. Wang and co-workers^47^ carried out a study of DA click reaction and formed a hydrogel under physiological conditions. Thanks to higher cross-linking density, the stretch and compress modulus of hydrogel reached 33.01 ± 4.50 kPa and 52.53 ± 3.16 kPa. Moreover, when the content of the antimicrobial peptide HHC10 was introduced via a thiol-ene click reaction more than 0.3 wt. %, the sterilization rate reached 100%.

#### 3. Amidation reaction (amide bonds)

**FIG. 3.**
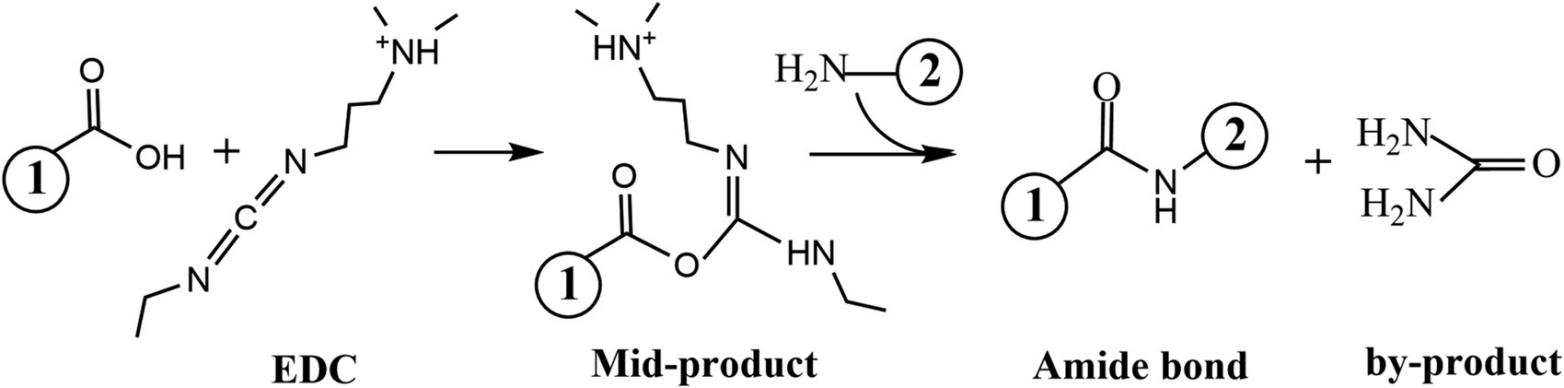
Formation process of the amide bond.

Amide bonds are formed by dehydration condensation reaction of carboxyl groups and amino groups; they are also called peptide bonds when multiple amide bonds exist at the same time.^48^ It can confer hydrogels with temperature sensitivity and adhesive property to tissues, for which reason they are often used in wound dressings. 1-ethyl-3-(3-dimethylaminopropyl)carbodiimide (EDC) and *N*-hydroxysuccinimide (NHS), which have been proved to be nontoxic and easily removed by dialysis or rinse, are often used in synthesis of amide bonds as catalysts to increase yields and decrease side reactions.^49,50^ As shown in Fig. 3, carboxyl groups were initially activated into a mid-product by EDC, and the amide bonds were formed in the reaction between the mid-product and compounds containing amino groups. The mid-product is unstable, while some irreversible and stable compounds would be formed in the process, so NHS was added to avoid the formation of the by-product.^51^

The carboxyl and amino groups involved in the reaction of amide bonds may be obtained from natural polysaccharides with a large number of groups and can also be provided by synthetic compounds. Singh *et al.*^52^ devised a hydrogel dressing to control bleeding, which exhibited a superior hemostatic property (CT, 225 ± 5 s). The novel hydrogel was fabricated by mixing of quaternized chitosan and phosphorylated chitosan, and tannic acid was added as adjuvant hemostat and a cross-linker. Pang *et al.*^53^ grafted 4-(4-(1-hydroxyethyl)-2-methoxy-5-nitrophenoxy) butyric acid (HMNB), a type of ortho-nitrobenzyl (ONB) molecule, onto PEG diamine to fabricate a wound dressing with UV-responsive and antibacterial ability. Zhang *et al.*^54^ synthesized carboxyl-modified PVA via esterification reaction between PVA and succine anhydride and prepared the hydrogel with chitosan. Hsu *et al.*^55^ reported a polypeptide/heparin composite hydrogel, which loads the growth factor to improve the effect for trauma treatment. The hydrogels based on linear and star-shaped poly L-lysine (l-PLL and s-PLL) can also be cross-linked by amide bonds, and compared to the hydrogels based on noncovalent interactions, the composite hydrogels have more stable structures and properties.

### B. Physical noncovalent cross-linking

The fabrication of hydrogels with chemical cross-linking reaction generally needs rigorous reaction conditions and the existence of cross-linking agents, while it is relatively easy to form physical conjugated hydrogels through host–guest interaction, electrostatic interaction, hydrogen bonding, hydrophobic interaction, and so on.

#### 1. Ionic interaction

Ionic interaction is a dynamic interaction between oppositely charged groups, or metal–ligand interactions, which can be used as an effective method for preparing hydrogels with the advantages of fast response to environmental stimulus and self-healing capability.^19^

The alginate-based hydrogel dressings are commonly synthesized by this technique, which rely on the divalent cations (e.g., Ca^2+^) as the cross-linking agents. Remaining polymers certainly can also be cross-linking via Ca^2+^ or other positive ions. A Pept-1/ alginate (ALG) nanocomposite hydrogel composed of a cell adhesive peptide conjugate (Pept-1) and a biocompatible alginate (ALG) was prepared via co-assembled and cross-linked with Ca^2+^.^56^ Gel-to-sol transition experiments of the nanocomposite hydrogel suggested the indispensable roles of ionic interaction during the co-assembling process. However, the full-thickness skin defect model of mice proved the Pept-1/ALG hydrogel could enhance adhesion and migration of fibroblast cells and accelerate wound heal rates.

Hydrogel obtained via ionic interaction tends to consist of two or more oppositely charged polymers. Polyelectrolytes like cellulose, gelation, and chitosan can easily form hydrogels via the attractive interaction between the negatively charged carboxyl groups and the positively charged amine groups or metal ions. These hydrogels could exhibit self-healing properties without any external intervention.^57^ The results of rheology showed a shear-thinning behavior of the chitosan-6-PG–Na^+^ hydrogel based on chitosan cross-linked with 6-phosphogluconic trisodium salt (6-PG–Na^+^) when increasing the shear rate,^58^ indicating that the hydrogel would be a candidate for injectable hydrogels. When the redox state or the coordination number of the metal center changed, the chitosan-6-PG−Na^+^ hydrogel undergoes sol–gel phase transition and is considered as a kind of stimuli sensitive hydrogel. The characteristics mentioned showed high probability of the hydrogels cross-linked by ionic interaction, which were used as wound dressings.^59^

#### 2. Hydrogen bonding

Hydrogen bonding is usually regarded as a weak interaction. Nevertheless, multiple hydrogen bonding is powerful to be used for the formation of hydrogel. In addition, hydrogen bonding would undergo dynamic destruction and reconstruction with the variation of temperature, pH, or solvent.^60,61^ Plenty of hydrogen donors and acceptors in natural polysaccharides, such as −OH, −COOH, and −NH_2_, could create the conditions for the formation of hydrogen bonding, so most of natural macromolecules containing gelatin, collagen, agar, and starch can form hydrogel by hydrogen bonding.^62–64^ Hydrogels cross-linked by hydrogen bonding alone are usually formed with some drawbacks, such as difficulty in injecting and weak mechanical strength due to their high content and low cross-link density. Therefore, it is suggested to combine the hydrogen bonding with other types of cross-linking techniques to obtain hydrogels with more outstanding properties.^65^

**FIG. 4.**
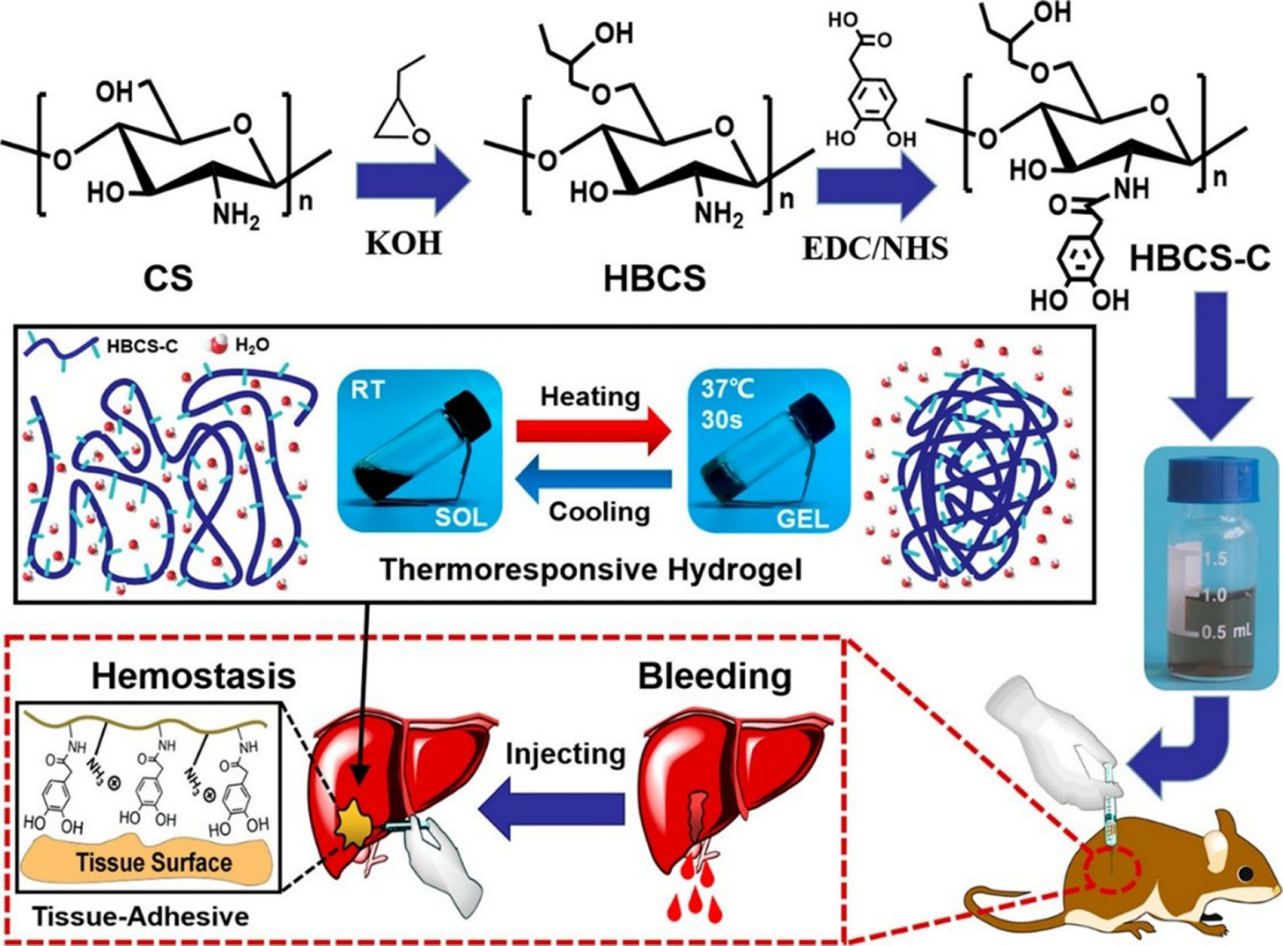
Schematic illustration of HBCS-C thermos-responsive adhesive hydrogel. Reproduced with permission from Shou *et al.*, ACS Biomater. Sci. Eng. 6, 3619–3629 (2020). Copyright 2020 American Chemical Society.^66^

In the work reported by Shou *et al.*,^66^ they prepared catechol-hydroxybutyl chitosan (HBCS-C) hydrogel, with the properties of hemostasis, thermo-sensitivity, injectable capability, tissue-adhesion, biodegradation, and biocompatibility, via hydrophobic interactions and hydrogen bonding (Fig. 4). Interestingly, because of the presence of the amino groups on CS backbones and the catechol groups introduced by amidation reaction, the HBCS-C hydrogel could firmly attach to skins or tissues by a synergistic interaction containing hydrogen bonding, π–π stacking, and cation–π interactions. As for the carboxyl polymer, the hydrogen bonding between polymer chains would be enhanced and their solubility wound be reduced at acidic aqueous solution; thus, they could form hydrogels by lowering the pH value of the aqueous solutions. For this reason, hydrogels relying on hydrogen bonding would exhibit pH-responsive performance.^67^

#### 3. Hydrophobic interaction

Due to the thermodynamic incompatibility of hydrophilic and hydrophobic moieties, amphiphilic polymers would self-assemble to form hydrogels by hydrophobic interaction in aqueous solutions.^68^ The hydrophobic interaction is a new strategy for the construction of biomedical hydrogels. Two main methods were involved to prepare hydrogels through hydrophobic interaction. The first one is to utilize micelles as cross-linking agents.^69^ Drugs can be incorporated into these copolymers for wound management. The second method is to graft hydrophobic moieties onto the hydrophilic precursor.^70^ The incorporation of hydrophobic groups into hydrophilic polysaccharide backbones provides the amphiphilic polymers with adaptive properties.

**FIG. 5.**
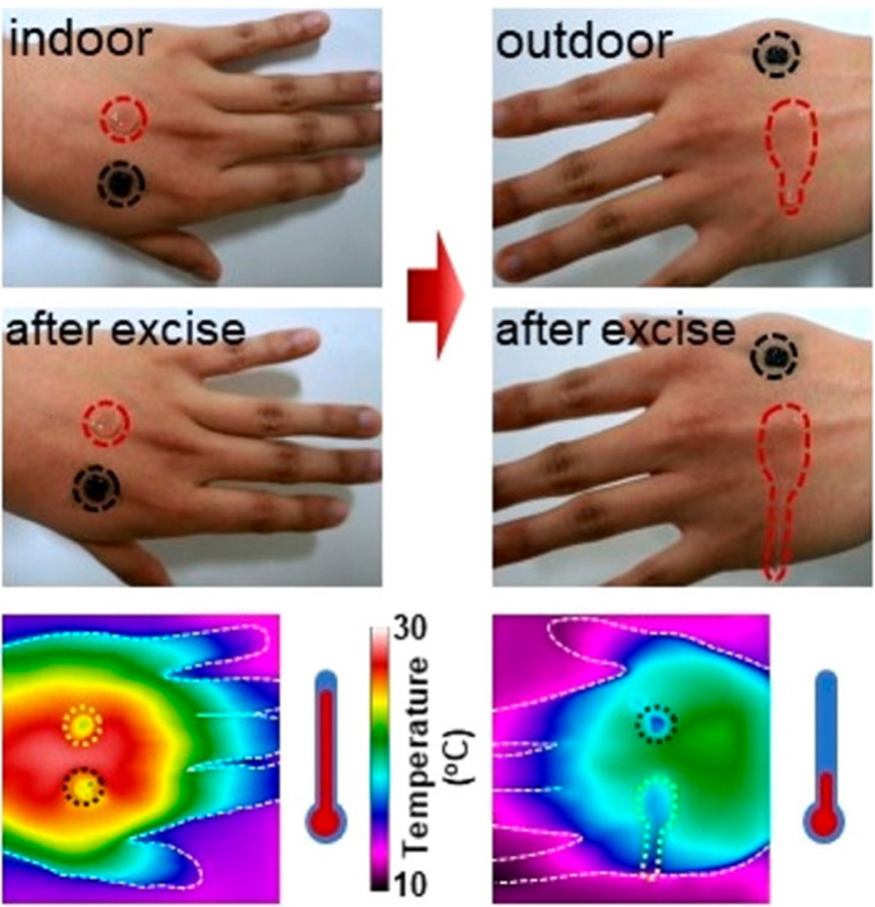
Digital pictures and infrared thermal images of the reversible rhodamine-PEP hydrogel and irreversible P(NIPAM_166_-*co*-nBA_9_)-PEG-P(NIPAM_166_-*co*-nBA_9_) graphene oxide nanosheets (PEP-AG) hydrogel on human hand skin indoors (warm) and outdoors (cold) in winter. Reproduced with permission from Yan *et al.*, ACS Nano **13**, 10074–10084 (2019). Copyright 2019 American Chemical Society.^72^

Hydrophobic interactions are frequently utilized to prepare thermos-responsive dressing, and the temperature sensitivity of hydrogels in aqueous solution relies on the delicate balance between hydrophilicity and hydrophobicity in a rational temperature range.^71^ Hydrogels as an *in situ* gel forming system, undergoing a sol-to-gel transition in a physiological temperature range (10–40 °C), have drawn intensive attention in the biomedical field. Some illustrative examples clearly demonstrate the feasibility of poly(ethylene glycol) (PEG), poly(*N*-isopropyl acrylamide) (PNIPAM), and so on in designing a thermo-gelling biomedical material.^72^ Yan *et al.*^72^ reported a sprayable *in situ* forming hydrogel as shown in Fig. 5. In order to obtain thermo-sensitive hydrogel response to the stimulus of body temperature, NIPAM served as thermo-sensitive A block of copolymers and the hydrophobic nBA were copolymerized to NIPAM, while PEG was selected as a hydrophilic B block to form hydrogel. As reported, the hydrogels that cross-linked via hydrophobic interaction generally exhibit excellent adhesive performance to tissues, especially living tissues under wet conditions, such as lungs and blood vessels.^73^ This property is beneficial for controlling bleeding and promoting wound healing. Nishiguchi *et al.*^74^ prepared monodisperse Alaska pollock-derived Gltn-microparticles (ApGltn-MPs) via self-assembly of gelatin in water–ethanol mixed solvents and thermal cross-linking. ApGltn with different hydrophobicity was fabricated via hydrophobic modification with aliphatic aldehydes that possess various chain lengths ranging from C6 to C12 and different adhesion strengths and stabilities were obtained.

## III. POLYMERS UTILIZED IN HYDROGEL WOUND DRESSINGS

The properties of the polymer almost determine the performances of the hydrogel made from this polymer. When preparing hydrogel wound dressings, our attention focused on biocompatibility, hemostatic properties, antibacterial properties, adhesion, and proper mechanical strength.^75–77^ To meet these demands, plenty of polymers are extensively utilized in the biomedical field including natural polysaccharide and synthetic polymers because of their attractive properties.

### A. Natural polymers

For their structure and properties of similar organisms, naturally polymeric materials have witnessed a real boom in recent years. Indeed, these natural polymers have already been developed for many biomedical applications, particularly in the field of hydrogel wound dressings.

#### 1. Collagen

Collagen is a natural structural protein with a unique triple-helical structure, which constitutes 25% of the total protein and is the most abundant protein in ECM proteins and the human body.^78^ It is produced by fibroblasts and involved in all three phases of the wound healing. Considerable evidence has indicated that collagen has the thrombogenic potential to stanch bleeding rapidly and repair various soft and hard tissues.^79,80^ The sources of collagen to make hydrogels are usually bovine, avian, or porcine, and collagen-based hydrogel dressings could create an environment that fosters healing and helps in cell migration and skin regeneration.^81^ Gelatin is a type of derivative of collagen, formed by breaking the natural triple-helix structure into single-strand molecules. It also has many biomedical applications for its biodegradability, biocompatibility, and the capability to encourage the organization and deposition of new collagen.^82^

Although collagen has excellent biological performance, its poor mechanical properties, high degradation rates, and inability to prevent bacterial growth limited its biomedical applications. Therefore, the production of collagen-based dressing is often accompanied by cross-linkers and other materials.^83^ Ding *et al.*^84^ introduced a method to generate a hydrogel dressing with injectable and rapid recovery capacity. They utilized dibenzaldehyde-modified PEG2000 (DA-PEG) as a cross-linking agent and constructed a collagen–chitosan (COL–CS) scaffold. The hydrogel showed good pH responsiveness due to the introduction of dynamic imine bonds. Thanks to the good adhesive ability, the COL–CS composite hydrogels had blood loss of 0.40 ± 0.15 g after 180 s in the mouse hemorrhaging liver model, which is excellent as compared to the 2.53 ± 0.19 g of the control group. According to the experimental processes and results, the reasons for the hydrogel dressing could be attributed to the wound surface as a network barrier might be the electrostatic interaction and hydrogen bonding between the polar groups on COL or CS backbones with the tissues.

As a derivative, gelatin is similar to collagen and can also act as a raw material for wound dressing for its outstanding biocompatibility and biodegradability. Chen *et al.*^85^ developed a multifunctional hydrogel with the unique properties containing self-healing, antibacterial, and sequential drug release. This hydrogel was constructed via the dynamic reaction of aminated gelatin (NGel), adipic acid dihydrazide (ADH), and oxidized dextran (ODex) under physiological conditions (pH 7.4). The hydrogel dressing exhibited excellent injectable capability because of the presence of dynamic imine bonds and acylhydrazone bonds. The results of the rat full-thickness skin wound model suggested that the novel hydrogel had fast stop bleeding and wound healing rate. Another derivative collagen peptide (COP) with good antioxidant properties is hydrolyzed from collagen. It is also particularly beneficial for hemostatic applications. As reported,^86^ Liu and his co-worker fabricated a novel hydrogel through the Schiff base reaction between the amino group from chitosan–collagen peptide (CS–COP) and the aldehyde group from oxidized konjac glucomannan (OKGM). Among them, CS–COP was obtained via COP grafted on the chitosan, under the catalysis of microbial transglutaminase (MTGase).

#### 2. Chitosan (CS)

CS is the only cationic polysaccharide in nature. Thanks to the presence of hydrophilic amino groups, chitosan could promote the adsorption of fibrinogen, thereby increasing platelet adhesion and thrombosis.^87^ Therefore, it is commonly used in hemostatic dressings. In addition, the chitosan used on the wound surfaces could promote cell proliferation, as well as collagen and hyaluronic acid (HA) formation and deposition.^88^ Regardless of all the above-mentioned benefits, low water solubility of CS and insufficient mechanical property and chitosan-based hydrogels limit the applications of CS. Therefore, it might be preferred to synthesize CS derivatives or modify different functional groups presented to overcome those limitations.^89^

**FIG. 6.**
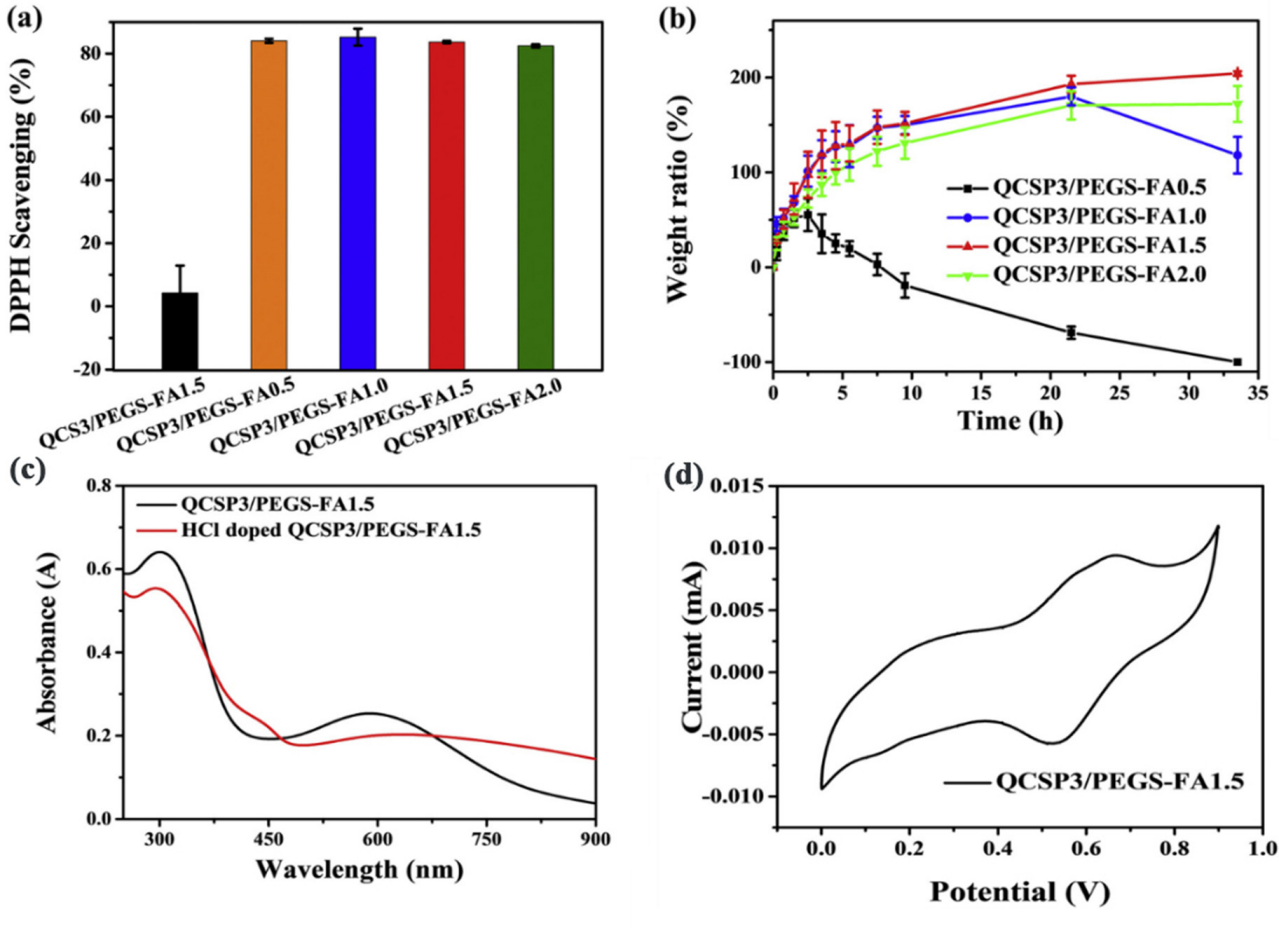
Characterization of the hydrogels: (a) antioxidant ability; (b) stability; and (c) and (d) electrochemical property. Reproduced with permission from Zhao *et al.*, Biomaterials **122**, 34–47. Copyright 2017 Elsevier.^94^

The modification of CS mainly relies on quaternization reaction between carboxyalkyl, hydroxyalkyl, or acyl derivatives with the amino groups of CS backbones, obtaining a new chitosan derivative with increased water solubility at a higher pH value.^90,91^ In addition, for improving the strength of chitosan-based dressing, CS be generally compounded with other polymers, including alginate,^92^ cellulose,^93^ and PEG,^94^ to form a complementary structure. Zhao *et al.*^94^ prepared quaternized chitosan through the reaction of PEG with the amino group of chitosan and further synthesized chitosan-g-polyaniline (QCSP) as the precursor. In addition, glycerin and ethylene glycol were added to improve the performance of gels. Chitosan-linked PEG can not only improve its performance but also eliminate the weak toxicity of the chitosan aldehyde group. An injectable hydrogel dressing with antioxidant, electroactive, self-healing, hemostasis, adhesiveness, and antibacterial ability was finally made via Schiff base reaction (Fig. 6).

Muzzarelli *et al.* proposed that glutaraldehyde is the most ideal cross-linker for preparing chitosan-based hydrogel.^95^ Chitosan-based hydrogel could be fabricated through the polymerization with glutaraldehyde in pH 4–5 lactic acid solution, at the molar ratio of amino groups and carbonyl groups to be around 10–20. Han and his co-workers^96^ synthesized polydopamine nanoparticles (PDA-NPs), which are nontoxic and no-aggregated near-infrared (NIR) absorbing agents. They prepared a series of different concentrations of PDA-NP precursors and then dispersed the precursors into the chitosan/silk fibroin mixed solution, and the CS/SF mixed solution was cross-linked through glutaraldehyde (1 wt. %) and freezing. The structure of this cryogel is similar to ECM, and the unique macroporous structure facilitated material exchange and cell attachment.

Apart from this, chitosan does not show any antibacterial activity at neutral pH, so some antibacterial agents were often incorporated to obtain chitosan-based dressing with antibacterial activity. Ehterami *et al.*^97^ utilized glutaraldehyde to cross-link chitosan and prepared a hydrogel dressing for dorsal skin injury. They added Alpha-tocopherol (vitamin E) to the hydrogel, evaluated the effect of the dosage of Vit E, and demonstrated the hydrogel could accelerate wound healing and cell proliferation. In addition, in the field of preventing scar formation, chitosan has unique advantages. Zhang *et al.*^98^ indicated that the chitosan hydrogel dressings would strongly affect the cell adhesion, proliferation, and differentiation through modulating the cross-linking degree and the cationicity (relatively available amine group) of chitosan. This advantage makes the chitosan-based dressing could prevent hypertrophic scar formation during wound healing process.

#### 3. Silk fibroin (SF)

As a natural fibrous protein derived from Bombyx mori, SF is composed of the tripeptide Arg-Gly-Asp (RGD) sequences, which contains one or more long chains of amino acid residues are soluble in water.^99,100^ The biodegradation products of SF are amino, which are helpful for angiogenesis, such as glycine and alanine and so on.^101,102^ SF is also beneficial to trauma treatment, which would participate in various phases of the healing process, and increase cell growth, adhesion, proliferation, and migration of different cell types.^103^ In addition, the strength and property of elastic recovery of hydrogel could be remarkably enhanced since the addition of SF.^104^ However, there are some drawbacks that restrict the biomedical applications of silk fibroin-based hydrogel, such as fragile and uneven.

Mehrabani *et al.*^105^ reported a chitin/silk fibroin/TiO_2_ bionanocomposite hydrogel dressing. In the experiment of the blood clotting, compared to control commercial dressing and pure blood, the hydrogel dressing showed better hemostatic ability. In addition, the composite hydrogel showed an ideal biodegradability; the recorded degradation was 30%–40% in the first week and reached 50%–60% and 60%–70% for second and third weeks, respectively. To obtain a hydrogel dressing with ideal mechanical and hemostatic properties, Wang *et al.*^106^ fabricated a cellulose/SF composite hydrogel (CSH) by a new CO_2_-incubation strategy. The result showed that the hemolysis ratio (HR) of all CSHs was lower than 5%, reflecting well hemocompatibility. In the hemostasis test, the blood loss and CT of CSHs were approximately 0.81 ± 0.10 g and 105.25 ± 21.55 s, respectively, superior to the performance of gauze (1.83 ± 0.26 g and 216.25 ± 31.02 s). After CO_2_-incubation treatment, the compressive strength of the CSH-0 hydrogel reached 2.53 ± 0.06 MPa, which can be attributed to the generation of hydrogen bonding between SF to make the matrix more aggregate. SF is often witnessed in the preparation of dressings for repairing infected wounds. SF was selected to fabricate an asymmetric wettable chitosan–silk fibroin [Chitosan (CTS)–SF/sodium alginate (SA)] dressing with loaded AgNPs. The porous structure of the dressing not only could absorb exudate but was also beneficial for the transmission of oxygen and nutrients into the wound.^107^

#### 4. Alginate

**FIG. 7.**
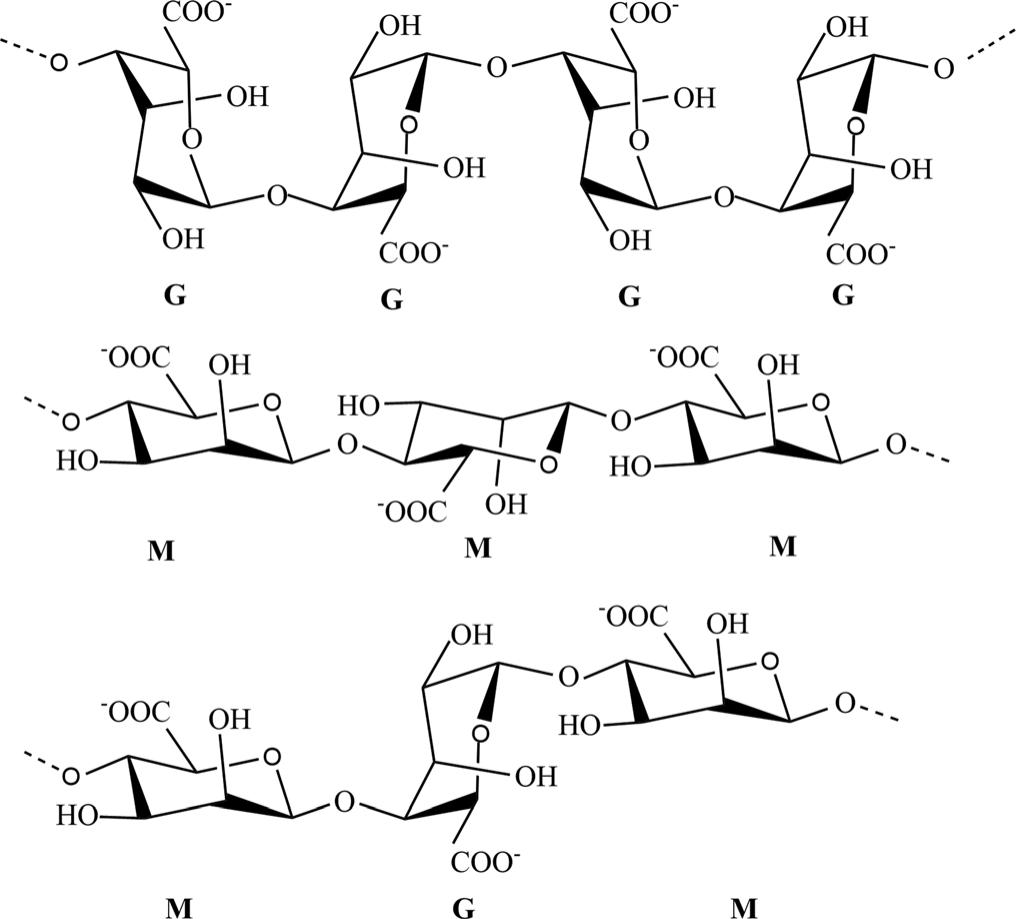
Chemical structures of the G-block, M-block, and alternating block in alginate.

Alginate is another natural polysaccharide with hemostasis and has been widely used as a hydrogel network component.^108^ As shown in Fig. 7, alginate is composed of D-mannuronic (often called M-block) and L-guluronic acid (often called G-block), wherein G-blocks are bent or distorted while M-blocks extended ribbon-like form.^109^ Alginate-based hydrogel dressings are generally prepared through the ionic cross-linking strategy of their solution with divalent cations, such as calcium, magnesium, barium ions, and zinc ions.^110–112^ There are only G-blocks that are able to participate in the gelation processes to produce hydrogels so that the M/G ratio and the number of repeated G-blocks in a matrix are important factors for physicochemical properties of ALG hydrogel.^111^ In general, hydrogel elasticity increases in the order GG (the alginate composed only by G-block) < MM (the alginate composed only by M-block) < MG (the alginate composed by M-block and G-block), while the order of strength is opposite.

**FIG. 8.**
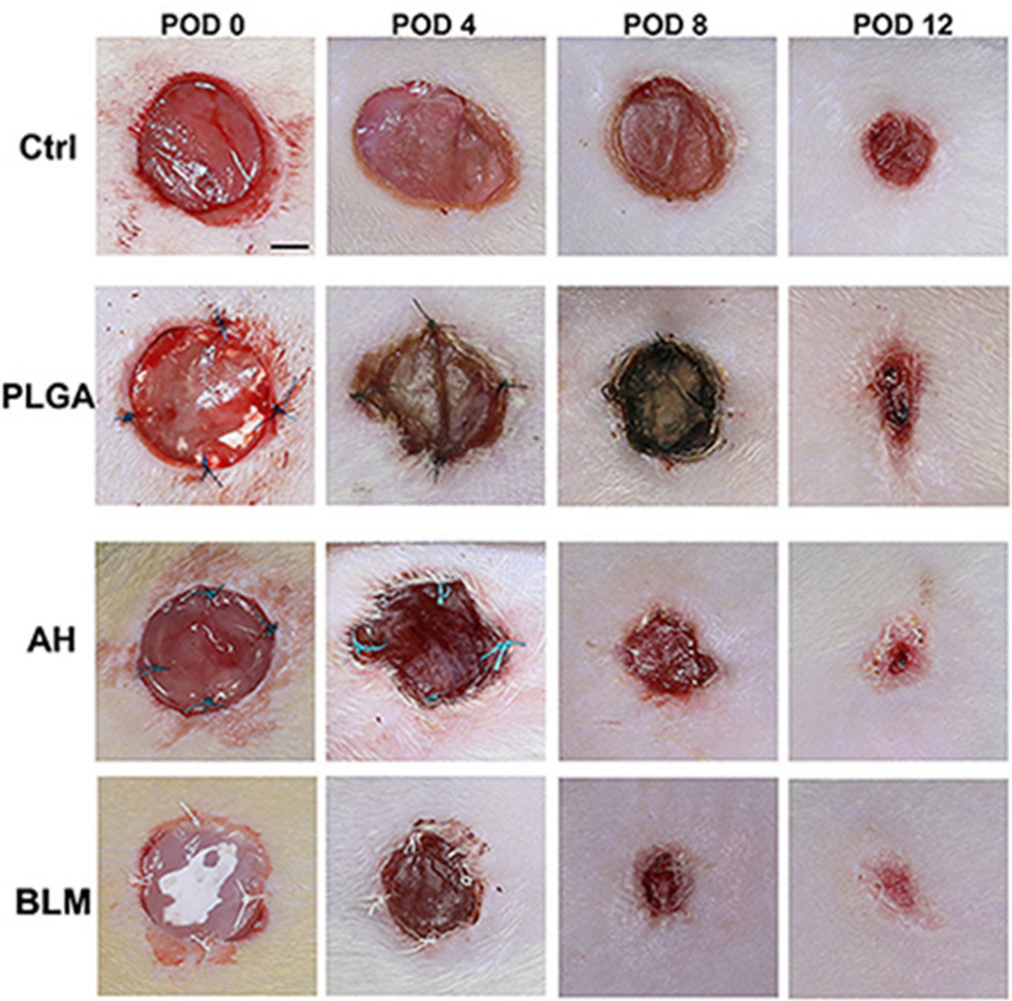
*In vivo* wound healing of wound treated by different dressings. Reproduced with permission from Wang *et al.*, Front. Bioeng. Biotechnol. **7**, 348 (2019). Copyright 2016 Authors, licensed under a Creative Commons Attribution (CC BY) license.^120^

CaCl_2_ is most commonly exploited as an ionic cross-linking agent of alginate hydrogels, and these hydrogels always form a fixed shape by the gel casting technique or solvent cast method and then cross-linked by immersing in CaCl_2_ solutions.^113^ In the gelation process of alginate-based hydrogels, the gelation is too quick to be controlled due to the high solubility of CaCl_2_ in the aqueous solution.^114^ In order to obtain hydrogels with uniform structures and greater mechanical integrity, CaSO_4_ and CaCO_3_ are in common usage instead. Catanzano *et al.*^115^ prepared a uniform and transparent alginate-based dressing with CaCO_3_ as the cross-linker. They also added HA and found the addition of HA could significantly decrease the gelation rate, while the concentration of cross-link is scarcely affected. Phosphate buffer saline (PBS) could also be used to increase gelation time, since the phosphate groups in the buffer and the carboxylate groups of the alginates compete the opportunity to react with calcium ions during the gelation process, and the gelation speed can be lowered.^116^ In addition, temperature is also an effective means of controlling the gelation process because of the limited reactivity of the divalent cations at low temperature. High gelation speed of alginate hydrogel is not all bad, since it is quite favorable for 3D bioprinted tissue constructs.^117^ Furthermore, the ability of alginate to increase the viscosity of precursor solution is beneficial to 3D printing technology, which means high shape fidelity. Markstedt *et al.*^118^ also perfectly combined the nanofibrillated cellulose (NFC) with the alginate and utilized advantages of the excellent shear-thinning properties and fast gelation speed to prepare a bioink, respectively. NFC as an effective strength additive also avoided the collapsing of the printing paste. As reported by Leppiniemi *et al.*,^117^ a nanocellulose–alginate composite hydrogel suitable for 3D printing was prepared. The hydrogel could be developed to be a generic platform to immobilize bioactive components, and the physical and chemical performances were improved due to the ionic cross-linking of Ca^2+^. The strength, compatibility, and absorption of the nanocellulose–alginate composite hydrogel suggested its potential in the field of biomedical applications, especially as wound dressings. Ilhan *et al.*^119^ printed a SA/PEG hydrogel and loaded *Satureja cuneifolia* plant extract (SC), which had a good effect on the treatment of diabetic wound as well as good antibacterial ability. Wang *et al.*^120^ designed a bilayer membrane (BLM) scaffold composed of poly (lactic-co-glycolic acid) (PLGA) and alginate. The alginate layer was fabricated on the surface of the PLGA membrane by 3D printing, which mimicked the dermis and could promote cell adhesion and proliferation *in vitro*. *In vivo* experiments demonstrated that the BLM scaffold could accelerate wound healing; the wounds treated with BLM scaffolds were completely healed by day 12, while 8.8%, 28.5%, and 33.7% of the wounds remained unhealed for the pure alginate hydrogel, PLGA, and the control groups, respectively (Fig. 8).

Thanks to its high hydrophilicity and re-gelation ability via absorbing exudate, alginate-based dressing is an ideal material for the treatment of moderate to heavily exuding wounds.^121^ However, natural alginate always shows limited biodegradation due to the lack of the enzyme that can cleave the molecular chains. Lee and Mooney believed that chemical modification of alginate through oxidation could control degradation.^109^ In addition, ionically cross-linked alginate-based hydrogels can be dissolved by releasing divalent ions; the process relies on the exchange reactions between the divalent cations in hydrogel and monovalent cations (e.g., sodium ions) in surrounding media. For this reason, sodium alginate (SA) and oxidized sodium alginate (OSA) oxidized with sodium periodate were more widely used compared with ALG.^122,123^

### B. Synthetic polymers

Although natural polymers are developed with many advantages, such as biocompatibility, biodegradability, low price, and a wide range of sources, they also have fatal shortcomings, especially difficult in modifying according to our demands. However, the synthetic polymer solves this problem very well.

#### 1. Poly(ethylene glycol) (PEG)

PEG is the synthetic compound with excellent biocompatibility certified by Food and Drug Administration (FDA). In addition, it also has other attractive properties including transparent, nontoxic, nonimmunogenic, and bioresorbable properties.^124,125^ Despite these, it is a relatively new material for preparing wound-dressing. The major classes of PEG can be distinguished as linear PEG and multiarmed star PEG (four-armed or eight-armed).^126–128^

Linear PEG is often used to prepare dressings with other polymers. Chen and his co-worker^129^ reported a hydrogel dressing, which was composed of CS and PEG and formed through the simple ester and amide linkages. The dressings could maintain a moist environment around the wound surface and provide suitable gas exchange volume. The results of *in vivo* experiment indicated the hydrogels have a prefect effect for both small cuts and full-thickness skin defects. In addition, they also found that PEG could enhance epithelial cell migration and promote wound healing. The linear PEG-based dressing can also consist of a triblock copolymer with a PEG middle block and other polymers' outer blocks or PEG outer block and other polymers' middle blocks.^130^ Gong *et al.*^131^ reported a poly(ethylene glycol)-poly(ε-caprolactone)-poly(ethylene glycol) (PCL-PEG-PCL) copolymer, which was synthesized through ring-opening polymerization and coupling reaction, and then prepared curcumin-loaded micelles. Due to the combination of the hydrophobic outer block and hydrophilic middle block, the triblock copolymer is amphiphilic in nature, and the sol–gel transition could happen at body temperature about 37 °C. According to the component and structure, some dressings consisting of pluronic F127 (PF127) may also be classified into this class.^132^

PEG can also be used as a raw material to form hyperbranched dendritic hydrogel. Ghobril *et al.*^133^ synthesized a lysine-based peptide dendrons with four terminal thiols. It could cross-link with poly(ethylene glycol disuccinimidyl valerate) (Succinimidyl valerate (SVA)–PEG–SVA) to form a hydrogel. This dressing based on thiol–thioester exchange for wound healing can provide effective protection for the wound to prevent secondary damage, thanks to its strong adhesion and ideal strength even after swelling in PBS. In addition, Moorcroft *et al.*^134^ designed a hydrogel/liposome system for the photothermal triggered release of antimicrobial agents.

**FIG. 9.**
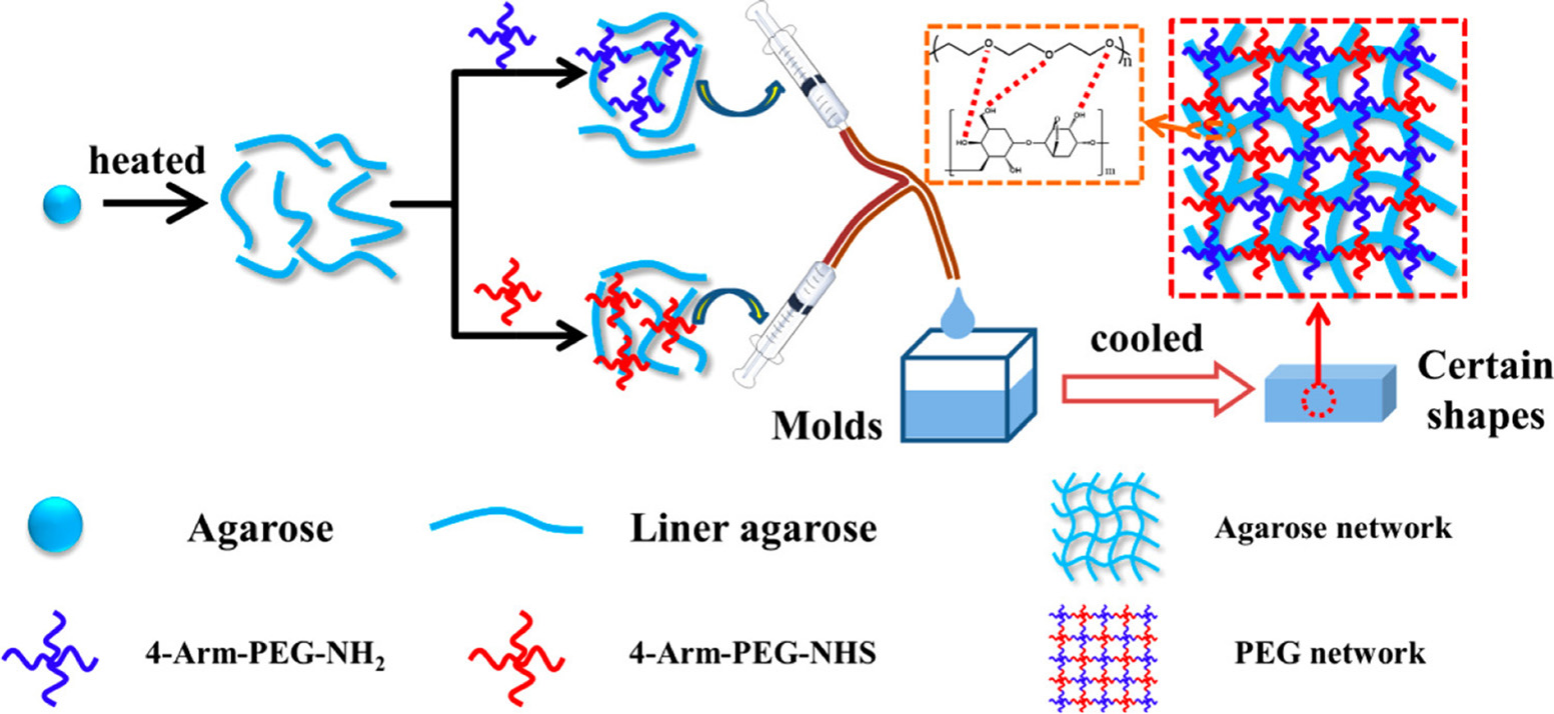
Schematic of the PEG–agarose DN hydrogel systems formed by mixing four-armed PEG–NH_2_ and four-armed PEG–NHS in 2% agarose solutions using a dual syringe. Reproduced with permission from Bu *et al.*, ACS Appl. Mater. Interfaces **9**, 2205–2212 (2017). Copyright 2017 American Chemical Society.^138^

The multiarmed star PEG could be used to prepare a kind of PEG-based dressing, the so-called PEG–PEG dressing. First of all, PEG-based polymers modified by different functional groups were synthesized.^135^ Subsequently, these functional PEG were mixed, and the hydrogel network was formed through the reaction between these groups. Bu *et al.*^136^ fabricated a vancomycin-loaded PEG-based hydrogel, which is a potential hemostatic and antibacterial dressing. First of all, four-arm-PEG–NH_2_, four-arm-PEG–NHS, and four-arm-PEG–CHO were synthesized, respectively, and then the hydrogel was formed via the reaction between these functionalized four-arm-PEG. In this study, the inherent drawback that the adhesion of PEG–PEG hydrogel to surrounding tissue is relatively weak could be addressed due to the formation of a Schiff base bond. In addition, the certified data showed that polyhedral oligomeric silsesquioxane (POSS)-modified PEG could reduce the hydrophilicity of PEG–PEG hydrogel.^137^ Bu *et al.*^138^ also reported PEG–agarose double network (DN) hydrogels (Fig. 9). It was formed via the reaction between four-arm-PEG–NHS and four-arm-PEG–NH_2_, and the hydrogen bond was generated by the heating/cooling of agarose. In addition, they also verified that the PEG-based hydrogel could be endowed fast degradable and controllably dissolvable properties after introducing cyclized succinyl ester groups,^139^ and eight-arm-PEG was employed for gelation, which could obtain a faster gelation rate than four-arm-PEG.^140^

#### 2. Poly(vinyl alcohol) (PVA)

Poly(vinyl alcohol) (PVA) is the most widely used polymer for fabricating hydrogels, especially hydrogel dressing. Similar to PEG, PVA is also biocompatible and capable of maintaining a moist environment.^141^ In particular, PVA is known for its antiprotein fouling and biologically inert properties.^142^ PVA-based hydrogel is a common physical cross-linked hydrogel, which could be produced by the freezing/thawing cycle and electron beam irradiation cross-linking strategy so that the usage of traditional chemical cross-linking agents and reagents can be avoided.^143,144^ In addition, PVA is generally combined with other materials or pharmaceuticals to form hydrogel dressing for a faster hemostasis wound healing rate.^145,146^

Chaturvedi *et al.*^147^ fabricated a PVA–ZnO nanocomposite through the repeated freezing/thawing approach and studied the influence of the PVA content and the number of freezing/thawing cycles. The results clearly indicated that the inherent porous structure of PVA hydrogel played a crucial role in swelling behavior and molecules going in and out through it. The swelling ratio of the composite hydrogel decreased with the increase in the PVA content and the number of freezing/thawing cycles, while the tensile strength showed the opposite pattern of change. Kuchaiyaphum *et al.*^148^ selected dialdehyde starch (DAS) as a cross-linker to prepare hydrogel dressing. The water vapor transmission rate (WVTR) of the hydrogel is approximately 2280 g m^−2^ day^−1^, which is suitable for maintaining the fluid balance on the wound interface and applicable to promote healing. Furthermore, the thermogravimetric analysis (TGA) result suggested that the thermal stability of the hydrogel could meet the demand of sterilization of biomedical applications.

For more excellent antibacterial properties and speeding up the wound healing, PVA-based dressing generally contains other materials or pharmaceuticals such as curcumin, zinc oxide nanoparticles, or sliver nanoparticles (AgNPs). Wang *et al.*^149^ designed a simple two-step process and fabricated PVA hydrogels containing Ag-doped TiO_2_ nanoparticles. The hydrogel showed excellent sterilization efficiency under visible light (VL) due to the presence of a small amount of Ag in TiO_2_. In addition, with the increase in silver concentration in Ag/TiO_2_, the generation of reactive oxygen species (ROS) shows a rising trend. The high-density hydrogen bonding formed between PVA and other polymers will delay the release of ROS, and this hydrogel can wipe out bacteria and promote repairing for a long time. However, the highly dense hydrogen bonds formed between the PVA and other polymers could hinder the release of the antibacterial agents and extend the effective time of the dressing. Fang *et al.*^150^ synthesized nonreleasing antimicrobial poly(ionic liquid)/PVA hydrogel dressing by both chemical and physical cross-linking, and it could controllably release positive charges. The poly(ionic liquid) was prepared through copolymerization between 1-vinyl-3-butylimidazolium bromide and acrylamide, and the existence of poly(ionic liquid) effectively could make up for poor antibacterial ability of PVA hydrogel. On the other hand, the stability of poly(ionic liquid) in hydrogel was also improved by compounding with PVA.

#### 3. Poly(N-isopropyl acrylamide) (PNIPAM)

**FIG. 10.**
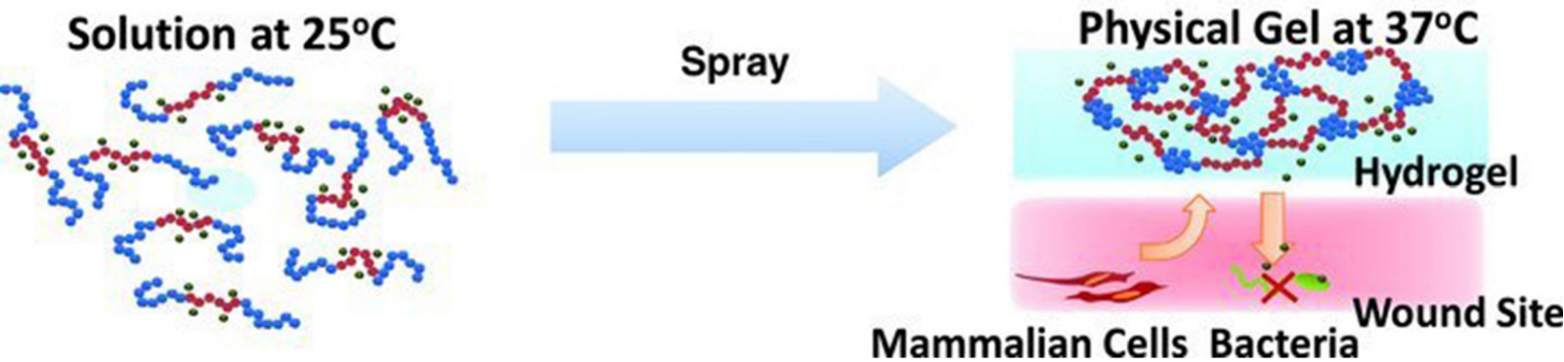
Schematic illustration of the *in situ* formation of antimicrobial PNIPAM-based dressing at the wound site. Reproduced with permission from Mi *et al.*, Adv. Funct. Mater. **21**, 4028–4034 (2011). Copyright 2011 John Wiley and Sons.^151^

It is well known that PNIPAM is a polymer that exhibits both hydrophobic and hydrophilic characteristics. PNIPAM exhibits hydrophilicity when the ambient temperature is below 30 °C and transforms into hydrophobicity above 35 °C. There is a delicate balance between the hydrophilic group (such as amide group) and hydrophobic group (such as isopropyl group) of PNIPAM chains around 32 °C. As exhibited in Fig. 10, this behavior is the so-called volume phase transition, and the temperature at which the transition occurs is called volume phase transition temperature (VPTT).^151–153^ In addition, microphase separation or macroprecipitation may occur when the system transforms from hydrophilic to hydrophobic, and the PNIPAM solution transforms from the homogeneous to heterogeneous system; hence, the temperature is also called lower critical solution temperature (LCST).^152^ Thanks to this reversible phase transition character, PNIPAM is often utilized to assemble thermo-sensitive hydrogel dressings.

Wu *et al.*^154^ used NIPAM that acted as a thermos-responsive drug release system and improved transparency and mechanical properties of hydrogel. Dong *et al.*^155^ designed a thermo-sensitive hydrogel composed of PNIPAM and poly(γ-glutamic acid) (PP), and it is loaded with superoxide dismutase (SOD) as drug to promote healing. As reported, Zubik *et al.*^70^ produced PNIPAM-based injectable hydrogels through free-radical polymerization and without any cross-linker. Due to the presence of NIPAM grafted onto the surface of CNCs and the resulting hydrogen bonding between metronidazole with NIPAM, the novel dressing was endowed the capability to release drugs stably and continuously at room temperature. Chen *et al.*^156^ produced a thermo-sensitive PNIPAM-based hydrogel dressing utilizing poly(amidoamine) (PAMAM) as a biocompatible cross-linking agent, and the hydrogel can also deliver the encapsulated drugs to designated tissues and organs. An *in vitro* investigation found that the hydrogel could control chronic inflammation and protease inhibiting activity, which promotes the secretion of TGF-β_1_ (transforming growth factor-β_1_) and β-FGF (β-fibroblast growth factor) and ultimately accelerate wound healing.

## IV. CONCLUSION AND PERSPECTIVE

Fatal bleeding and wound repair have caused a great deal of burden. Despite the fact that bandages, gauzes, and sutures remain the common wound closure techniques, they are inefficient in preventing infection and accelerating wound healing. In order to address these issues, much effort has been adopted for the research and development of novel and efficient dressings over recent years. Hydrogel, which exhibits a 3D network structure with a lot of attractive features (e.g., high moisture, sufficient strength, superior elasticity, and easy to be modified), has been popularly used as wound dressings. Hydrogel can be prepared by different cross-linking strategies and classified into different ways based on their constituents. We have discussed the commonly used cross-linking techniques and the composition for the manufactures of hydrogels as wound dressings. As mentioned above, polysaccharide-based hydrogels possess intrinsic characteristics such as biocompatibility, biodegradability, and nontoxicity. However, synthetic polymer-based hydrogels can be easily modified and have better mechanical property.

However, there are still some obstacles needed to be overcome. In some cases, it is unavoidable for using cross-linking agents or other additives for developing matrix or gaining better performance of hydrogels. It should be confirmed that all the introduced components are not harmful to health. In the future work, fabricating nontoxic and degradable dressing will the first issue to be addressed, which would rely on the development of a new cross-linker, various cross-linking techniques, and modification methods of polymers. Second, hemostatic and antibacterial performance are critical evaluation standards for wound dressing. In addition, in order to achieve effective prevention of infection or promotion of wound repair, specific bioactive species (e.g., antibiotics and growth factor) or other antibacterial ingredients (e.g., ZnO nanoparticles and Ag nanoparticles) should be added to the dressing. Furthermore, improving the comprehensive properties of dressing, for instance, mechanical behavior, swelling capacity, and tissue adhesion, is significant. Some novel strategies have been proposed to meet these demands, such as hydrogels with DN/interpenetrating polymeric network (IPN) structures showed good mechanical property, and mussel-inspired hydrogel presented enhanced adhesive performance. Moreover, exploring smart hydrogel dressings would be the main signpost for the future advancement of this field. It is desired to develop hydrogels with properties like shear-thinning behavior, sensitivity to external stimulus, self-healing property, etc. Particularly, the weakly acid environment of wound could provide an effective way to trigger sol–gel transition of pH-responsive hydrogel, which would be beneficial to *in situ* gelation and removal. Interdisciplinary integration of polymer synthesis with molecular biology, tissue engineering, and bioelectrochemistry might be an effective approach to obtain smart wound dressing. All aforementioned features represent opportunities for the design of novel hydrogels. However, considering the clinical application, controllable costs and simplified manufacturing method are also one of the research directions in the laboratory.

There are some factors that may influence the characteristic of hydrogel wound dressing. Therefore, the design and fabrication of hydrogels for wound dressing should be considered comprehensively, including meaningful multifunction, improvement of existing performances, stability of in all aspects, care impact for wound, and processability. It is conceivable that the current challenges will be resolved and hydrogel dressing will be a promising candidate for wound healing in near future with the continuous effort in this field.

## Data Availability

Data sharing is not applicable to this article as no new data were created or analyzed in this study.
